# Preliminary Exploration of *MAGE-B1*, *-B4*, *-B5*, and *-B10* mRNA Expression in Canine Mammary Tumors in Dogs

**DOI:** 10.3390/ani15070910

**Published:** 2025-03-21

**Authors:** Wanwisa Srisawat, Pongpisid Koonyosying, Anucha Muenthaisong, Kanokwan Sangkakam, Thanya Varinrak, Nattawooti Sthitmatee

**Affiliations:** 1 Laboratory of Veterinary Vaccine and Biological Products, Faculty of Veterinary Medicine, Chiang Mai University, Chiang Mai 50100, Thailand; wanwisasrisawat@gmail.com (W.S.); pongpisid.koo@cmu.ac.th (P.K.); anucha.m@cmu.ac.th (A.M.); kanokwansangkakam@gmail.com (K.S.); thanya.var@cmu.ac.th (T.V.); 2 Multidisciplinary Research Institute, Chiang Mai University, Chiang Mai 50100, Thailand; 3 Office of Research Administration, Chiang Mai University, Chiang Mai 50200, Thailand; 4 Center of Veterinary Medical Diagnostic and Animal Health Innovation, Chiang Mai University, Chiang Mai 50100, Thailand; 5 Research Center for Veterinary Bioscience and Veterinary Public Health, Chiang Mai University, Chiang Mai 50100, Thailand

**Keywords:** canine mammary tumors, melanoma-associated antigen-B, mRNA expression, quantitative real-time PCR

## Abstract

The melanoma-associated antigen gene (MAGE) is a key target in cancer immunotherapy. Given the potential of MAGE-B genes in veterinary immunotherapy for canine mammary tumors (CMTs), this study investigated the mRNA expression of *MAGE-B1*, *-B4*, *-B5*, and *-B10* in CMT tissues and cells from dogs. Quantitative real-time PCR was used to analyze 28 CMT tissue samples, including 4 benign and 24 malignant tumors (13 simple carcinomas, 6 complex carcinomas, 3 carcinosarcomas, and 2 fibrosarcomas). Benign mixed tumor and complex carcinoma-type CMT cells were cultured and treated with a DNA methylase inhibitor (5-aza-2′-deoxycytidine; 5-aza-CdR) and a histone deacetylase inhibitor (Trichostatin A; TSA) under the following four conditions: (1) 5-aza-CdR for 72 h; (2) TSA for 24 h; (3) 5-aza-CdR for 48 h followed by TSA for 24 h; and (4) control. *MAGE-B1* and *-B4* showed the highest expression in the CMT samples (100% and 89.29%, respectively), followed by *MAGE-B10* (82.14%). Carcinosarcomas and simple anaplastic carcinomas had significantly higher MAGE-B expression levels than simple tubulopapillary carcinomas (*p* < 0.05). 5-aza-CdR treatment increased MAGE-B expression, whereas TSA had a mild effect. Further research involving larger cohorts is needed to confirm these findings.

## Simple Summary

The melanoma-associated antigen gene (MAGE) is an important target for cancer immunotherapy. *MAGE-B* genes may be involved with the tumor progression biomarkers of canine mammary tumors (CMTs) and have been used as immunotherapy target genes for cancer vaccines. Thus, this study looked at the mRNA expression of *MAGE-B1*, *-B4*, *-B5*, and *-B10* in CMT tissues and cells from dogs. Researchers examined 28 tumor samples (4 benign and 24 malignant) using a real-time PCR technique. They also grew CMT cells and treated them with two reagents—a DNA methylation inhibitor (5-aza-2′-deoxycytidine; 5-aza-CdR) and a histone deacetylase inhibitor (Trichostatin A; TSA)—under four different conditions. The results showed that *MAGE-B1* and *-B4* had the highest mRNA expression in the CMT samples (100% and 89.29%), followed by *MAGE-B10* (82.14%). Tumors classified as carcinosarcomas and simple anaplastic carcinomas had significantly higher *MAGE-B* mRNA expression levels than simple tubulopapillary carcinomas (*p* < 0.05). Cells treated with 5-aza-CdR showed increased *MAGE-B* mRNA expression, while TSA had little effect. A larger study is needed to confirm these findings and better understand *MAGE-B* mRNA expression patterns.

## 1. Introduction

Canine mammary gland tumors (CMTs) are the most common type of tumor in female dogs [1,2,3]. The incidence of CMTs varies by country [4,5,6]. Surgery, involving the removal of tumors with clean margins, is the primary treatment method for the control of CMTs other than for inflammatory carcinoma, where palliative medical treatment and chemotherapy are preferred [7,8,9]. In unspayed dogs, ovariohysterectomy (OHE) is also performed at the same time as surgery [10,11]. However, recent studies have shown that not all CMT cases benefit from OHE [12]. Unfortunately, in advanced cancer, tumor cells may remain in the adjacent tissues after surgery, leading to tumor recurrences [13,14,15,16]. Chemotherapy and radiotherapy are subsequently performed in these cases [10,16]. In addition, alternative treatments, such as immunotherapy, including tumor vaccines and targeted monoclonal antibodies, hormonal therapy specific to hormonal biomarker expression, and metronomic therapy, provide new hope to improve patients’ quality of life [9,17]. However, one promising strategy of immunotherapy involves the stimulation of the function of the lymphocytes, mainly the cytotoxic T lymphocytes and natural killer cells, which act in response to the eradication of cancer cells as a result of vaccines against tumor-remaining cells after surgery; this strategy is limited in relation to dogs [18].

*Melanoma-associated antigen genes (MAGEs)* were first discovered in human melanoma cell lines [19]. Later, *MAGEs* were also demonstrated in many other cancer cell types [20,21,22,23,24]. In general, *MAGEs* are divided into two types based on their location on the chromosome [25,26]. Type I *MAGEs* are normally located on the X chromosome, and their protein is classified in the cancer–testis antigen category [27]. Type II *MAGEs* exhibit variable expression levels and are located on non-X chromosomes. Their protein is reported in various tissue types [28]. Type I MAGEs include MAGE-A, -B, and -C, whereas Type II MAGEs include MAGE-D, -E, -F1, -G1, -H1, L2, and Necdin [26,29].

*MAGE-B* genes are found in various types of human tumors, including melanoma, non-small-cell lung carcinoma, sarcoma, esophageal squamous cell carcinoma, colorectal cancer, and mammary carcinoma [30,31,32,33]. MAGE-B-based vaccines have been developed to combat the metastasis of advanced mammary tumors in mouse models [34,35,36]. Moreover, studies on MAGE-B-based vaccines using recombinant *Listeria monocytogenes* as a vector have demonstrated effectiveness against both primary tumors and metastasis mammary tumors in mouse models [37,38,39]. These findings highlight that MAGE-B genes have promising potential for immunotherapeutic study in relation to veterinary oncology.

In chromosomal regions of tumor-associated genes, epigenetic modifications may constitute important regulatory mechanisms for the pathogenesis of tumor transformation [40]. Previous studies in humans have shown that *MAGE-A* gene expression is regulated by DNA methylation and histone deacetylation, as its expression can be induced by 5-aza-2′-deoxycytidine (5-aza-CdR) and Trichostatin A (TSA) in cells where MAGE-A gene expression is normally silent [41,42,43,44]. Additionally, regulating *MAGE-C1* and *-C2* has been suggested to involve DNA methylation, as their gene expression was induced by 5-aza-CdR [45]. However, the regulatory mechanisms governing *MAGE-B* gene expression in tumors are not fully understood.

Therefore, the objectives of the present study were to detect *MAGE-B1*, *-B4*, *-B5*, and *-B10* mRNA expression levels in CMT tissues by employing a quantitative real-time PCR (qPCR) method and to evaluate the epigenetic mechanism involved with their expression using 5-aza-CdR and TSA reagents.

## 2. Materials and Methods

### 2.1. Tissue Samples

Twenty-eight fresh tumor tissue samples of dogs diagnosed with canine mammary gland tumors that were admitted for surgical removal mastectomy at Chiang Mai University Small Animal Teaching Hospital were collected under the owner’s permission between November 2017 and December 2018. Twenty-eight tissue samples were used for total RNA extraction and were gently cut and equally separated into two parts. The first part of each sample was kept in 4% formaldehyde-buffered solution for histopathological classification. The pathological results were analyzed at the Center of Veterinary Medical Diagnostic and Animal Health Innovation, Chiang Mai University, according to the histologic classification: 2010 [46]. The second part of each sample was immediately frozen with liquid nitrogen and stored at −70 °C. The samples included 4 benign mixed tumors, 13 carcinoma-simple, 6 carcinoma-complex, 3 carcinosarcomas, and 2 fibrosarcomas. Two normal testicular tissue samples were obtained from dogs admitted for castration. The study was conducted according to the guidelines of the Institute of Animals for Scientific Purposes Development (IAD) and the National Research Council of Thailand (NRCT); it was approved by the Animal Care and Use Committee (FVM-ACUC), Faculty of Veterinary Medicine, Chiang Mai University (protocol code: S30/2560; date of approval: 19 January 2018).

### 2.2. Primary Cell Culture

Two CMT primary cells consisting of one benign mixed tumor and one carcinoma- complex type (passage 5) were obtained from Fhaikrue and colleagues; the cells were expanded in our laboratory [47]. Briefly, 100 g of fresh tissue sample was gently minced on ice under aseptic conditions. The tissues were washed three times with 1% penicillin/streptomycin (Gibco™, Thermo Fisher Scientific, Waltham, MA, USA) in sterile phosphate-buffered saline (PBS) and then trypsinized in 0.25% trypsin in Dulbecco’s Modified Eagle Medium (DMEM; Gibco™, Thermo Fisher Scientific) with 1% penicillin/streptomycin at 4 °C for 16–18 h. The tissue was incubated and shaken at 37 °C for 30 min. The cell pellets were collected and resuspended in 10% fetal bovine serum (FBS; Gibco, Waltham, MA, USA) in 10 mL of medium. The cell solution was then filtered using the 70 µm nylon cell strainer. The filtered cell pellets were resuspended in 10 mL of 10% FBS medium. Finally, the target cells were transferred into 25 cm^2^ flasks and cultured at 37 °C under 5% CO_2_. Subculturing was performed when cells reached over 80% cell confluence. Cells were enumerated for an appropriate density. Subculturing and cell density estimation were performed every 3 days, with the process repeated until passage 5. Then, cuboidal and spindle-shaped CMT-diagnosed cells were confirmed using the immunoperoxidase monolayer assay (IPMA), for which both of them reported strong positivity with mouse anti-panCK mAb (clone AE1+AE3, Diagnostic BioSystems, Pleasanton, CA, USA), weak positivity with mouse anti-vimentin mAb (clone V9, Diagnostic BioSystems), and negativity with rabbit anti-FAP pAb (clone 53066, Abcam, Cambridge, UK).

### 2.3. 5-aza-CdR and TSA Treatment of Cells

The protocol of the 5-aza-CdR and TSA treatment of cells was adapted from the method of Wischnewski and colleagues [48]. Briefly, each primary cell culture was counted using trypan blue solution (Invitrogen^TM^, Thermo Fisher Scientific). The cells were then split into 4 groups with each flask containing approximately 1.7 × 10^5^ cells and 2.5 mL of cultured media. The cells were incubated at 37 °C under 5% CO_2_ for approximately 48 h until they reached 40–50% confluence. Before treatment, the cells were washed once with DMEM. The treatment conditions were as follows: (1) 5-aza-CdR group—cells were stimulated by 1 µmol/L 5-aza-CdR (ab1208412, Abcam Limited, Cambridge, UK) for 72 h; (2) TSA group—cells were stimulated by 1 µmol/L TSA (ab120850, Abcam Limited, Cambridge, UK) for 24 h; (3) 5-aza-CdR and TSA group—cells were stimulated by 1 µmol/L 5-aza-CdR for 48 h and were subsequently stimulated by 1 µmol/L TSA for 24 h; (4) control group—cells were treated with culture medium in place of reagents, in equal volume.

### 2.4. Total RNA Extraction of Tissue Specimen

Liquid nitrogen was immediately poured over the frozen tissue samples, and they were ground using an RNAse-free mortar and pestle until they became powder-like. RNA extraction was performed using the TRIzol reagent (Invitrogen^TM^, Waltham, MA, USA) by following the manufacturer’s instructions. Extracted RNA samples were quantified using a UV/Vis spectrophotometer (Beckman coulter, Brea, CA, USA), and an absorbance ratio of 260/280 was measured to determine RNA purity. RNA samples were then assessed with RNA electrophoresis using ethidium bromide staining to check the integrity of the samples. The degraded RNA specimens were excluded from analysis [49].

### 2.5. Total RNA Extraction of Cell Culture

The culture media were discarded, and the cultured cells were gently washed three times with cold phosphate-buffered saline (PBS). Total RNA was extracted using an RNA extraction kit (Invitrogen^TM^) under cold conditions, according to the manufacturer’s instructions. The extracted RNA samples were quantified using a UV/Vis spectrophotometer (Beckman coulter), and an absorbance ratio of 260/280 was measured to determine RNA purity.

### 2.6. cDNA Synthesis

cDNA was synthesized using the reverse transcription enzyme according to the manufacturer’s instructions (Invitrogen^TM^). Briefly, 4.5 µg of total RNAs was used as the template in the 20 µL cDNA synthesis reaction. The total RNAs were mixed with 50 µM oligo (dT) 20 primer, 10 mM dNTP mix, and DEPC-treated water; then, the mixture was incubated at 65 °C for 5 min, and immediately chilled on ice. A mixture of 10× RT buffer, 25 mM MgCl_2_, 0.1 M DTT, 40 U RNase inhibitor, and 200 U reverse transcriptase enzyme was added to the reaction, which was then incubated at 50 °C for 50 min, followed by incubation at 85 °C for 5 min, and immediate cooling on ice. RNase H was then added to the reaction and incubated at 37 °C for 20 min. The cDNA samples were stored at −20 °C until being used.

### 2.7. qPCR Methods

#### 2.7.1. Primer Design

Specific primer pair sequences for the canine *MAGE-B1*, *-B4*, *-B5*, and *-B10* genes and canine *GAPDH* gene were designed using Oligo 7 Primer Analysis Software (Molecular Biology Insights, Colorado Springs, CO, USA). The primer sequences were selected to be specific to the coding region of the targeted genes. The primers were designed to span two exons, targeting the regions of the gene that encode the final mature mRNA after the introns had been removed via RNA splicing. Each primer pair was confirmed to have no homology to other dog sequences using the Primer-BLAST tool at https://www.ncbi.nlm.nih.gov/tools/primer-blast/, accessed on 10 July 2018.

#### 2.7.2. Primer Selection

PCR amplifications were performed using an RBC *taq* DNA polymerase kit (RBC Life Sciences Inc., Irving, TX, USA) according to the manufacturer’s instruction. Each amplification utilized 50 ng of the total RNA sample extracted from testicular tissue as a control, which was directly compared to the cDNA. The cycling conditions were as follows: initial denaturation at 94 °C for 5 min; 35 cycles of denaturation at 94 °C for 30 s; annealing at a gradient of 55–60 °C of MAGE-B1, -B4, -B5, and -B10, as well as GAPDH primers for 30 s; extension at 72 °C for 30 s; and a final extension at 72 °C for 10 min. The PCR products were electrophoresed on 2% agarose gels stained with Maestrosafe nucleic acid loading dye (Maestrogen, Hsinchu, Taiwan). Electrophoresis was performed for 30 min at 100 V to check the product size by comparing it with a DNA molecular size marker. The bands were visualized under UV (UVP Benchtop UV Transilluminators; Fisher Scientific, San Francisco, CA, USA). A single amplified fragment was purified using a PCR purification kit (Invitrogen^TM^) following the manufacturer’s suggested protocol. Direct sequencing analysis was performed to confirm the homology of the PCR products. Sequence identification and multiple alignment analysis were conducted using the BLAST program (http://blast.ncbi.nlm.nih.gov, accessed on 17 August 2018) of the National Center for Biotechnology information (NCBI), and multiple sequence comparison was performed using log-expectation (MUSCLE).

#### 2.7.3. Standard Curve and Regression Analysis for qPCR Conditions

qPCR was performed using serial dilutions, which were composed of 100 ng, 20 ng, 4 ng, 0.8 ng, 0.16 ng, 0.082 ng, 0.064 ng, and 0 ng of the total RNA sample extracted from testicular tissue as a control, which was directly compared to the cDNA. Each condition was tested in triplicate. The average Ct values from triplicate measurements were calculated for each dilution of the sample. A coefficient of determination (R^2^) ≥ 0.95 was considered acceptable. The amplification efficiency was calculated using the slope of the linear regression with the following formula:

$$\mathrm{Amplification}\;\mathrm{efficiency}=\left[10^{-\left(\frac{1}{Slope}\right)}\right]-1$$

#### 2.7.4. Sensitivity Evaluation of Real-Time PCR Method

The appropriate number of samples was analyzed using the total RNA extracted from the testicular sample, which was converted to cDNA in five different dilutions. Briefly, serial dilutions with 5-log dilutions of the sample were prepared, including 400 ng, 80 ng, 16 ng, 3.2 ng, 0.64 ng, 0.128 ng, 0.0256 ng, and 0 ng. The qPCR reaction using GAPDH primer pairs was performed in triplicate. The amount of sample required for detecting mRNA expression using the qPCR assay was evaluated.

#### 2.7.5. qPCR Conditions

qPCR reactions were conducted in a 96-well plate using the ABI PRISM 7000 Real-Time system (Applied Biosystems^TM^, Thermo Fisher Scientific, Waltham, MA, USA). Each reaction was performed in duplicate with 20 µL of qPCR reaction mixture prepared using 25 µg/2 µL of cDNA sample, 10 µL of 2×SensiFAST SYBR^®^ No-ROX Mix (Meridian Bioscience, Memphis, TN, USA), and the specific primers according to the manufacturer’s recommendation. The amplification cycle began at 95 °C for 2 min, followed by 40 cycles at 95 °C for 15 s, 57 °C for 15 s, and 72 °C for 20 s. The dissociation step was performed at 95 °C for 15 s, 60 °C for 15 s, and 95 °C for 15 s. The Ct cycle was recorded.

### 2.8. Data Analysis

A descriptive analysis was used to explain the results of this study. mRNA expression was considered positive if the Ct cycle was ≤35; otherwise, it was grouped as no mRNA expression. The proportion of MAGE-B mRNA expression was calculated for each type of tumor. The relative mRNA expression of *MAGE-B* target genes, in comparison to *GAPDH* (a housekeeping gene), was calculated using the 2^(−∆∆CT)^ method [50]. The mean level of relative mRNA expression in CMT tissues was presented using descriptive analysis, and the levels were compared between benign and malignant CMTs using an unpaired *t*-test due to the unequal sample size between groups. Among malignant CMTs, the mean level of relative mRNA expression between groups was compared using a nested *t*-test. A comparison of mRNA expression levels between CMT culture cells in different groups was performed using one-way analysis of variance (ANOVA). All the statistical analyses were performed using GraphPad Prism 9.4.0 (Insight Partners, New York City, NY, USA). A statistically significant difference was accepted at *p* < 0.05.

## 3. Results

### 3.1. Primer Design, Specificity, and Sensitivity

The results showed that specific primer pairs were established for a quantitative real-time PCR method for the detection of *MAGE-B1*, *-B4*, *-B5*, and *-B10* mRNA expression in canine mammary tumors (CMTs), as shown in Table 1. The R^2^ value of qPCR methods using *GAPDH*-, *MAGE-B1*-, *MAGE -B4*-, *MAGE -B5*-, and *MAGE -B10*-specific primer pairs ranges from 0.97 to 0.99. The amplification efficiencies of the methods were 0.90, 0.92, 0.93, 0.88, and 0.98, respectively. The sensitivity test showed that approximately 25 µg of total RNA, which had already converted to cDNA, was acceptable for use in the present assay. The average Ct values for the sample at 16 ng and 80 ng were 18.24 ± 0.11 and 15.76 ± 0.06 for *GAPDH* mRNA expression in testicular tissue samples, respectively.

### 3.2. The Proportion of MAGE-B1, -B4, -B5, and -B10 mRNA Expression in CMTs

In the present study, the proportion of *MAGE-B1* and *MAGE-B4* mRNA expression in CMTs was highest compared to other *MAGE-B* genes, followed by *MAGE-B10* and *-B5*. The proportions were 100% (28/28), 85.7% (24/28), and 82.1% (23/28), respectively (Table 2). When analyzed according to gene among malignant CMTs, *MAGE-B1* and *-B4* mRNA expression was present in all types of CMTs, with proportions of 100% (24/24) for each gene. Additionally, the proportion of *MAGE-B10* mRNA expression was highest in carcinosarcomas, followed by epithelial neoplasms and mesenchymal neoplasms, with a proportion of 100.0% (3/3), 68.4% (13/19), and 50% (1/2), respectively. The proportion of *MAGE-B5* mRNA expression was highest in epithelial neoplasms, followed by carcinosarcomas and mesenchymal neoplasms, with proportions of 84.2% (16/19), 75.0% (2/3), and 50% (1/2), respectively. In benign CMTs, *MAGE-B1*, *-B4*, *-B5*, and *-B10* mRNA expression was observed in all types of CMTs.

### 3.3. The Relative mRNA Expression of MAGE-B1, -B4, -B5, and -B10 in CMT Tissues

**Figure 1:**
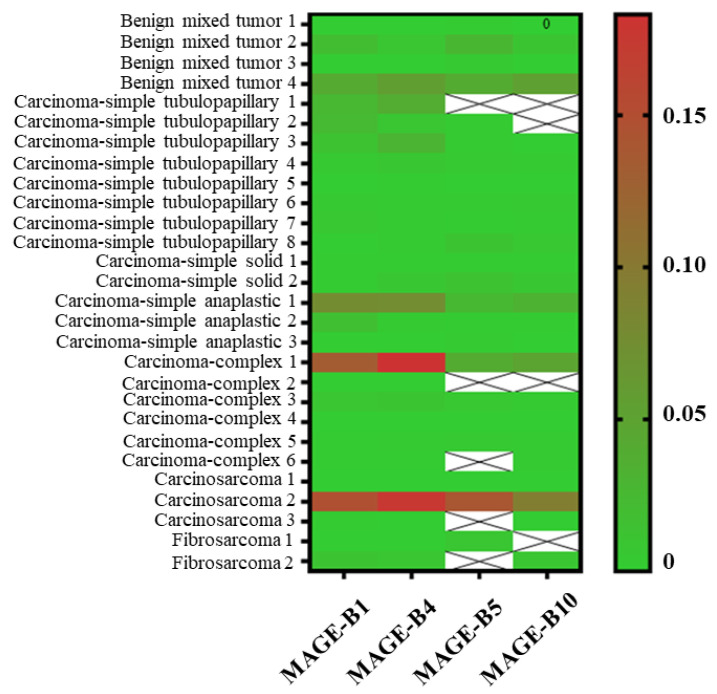
The relative fold change in the mRNA expression of *MAGE-B1*, *-B4*, *-B5*, and *-B10* from canine mammary tumor tissues using *GAPDH* as a reference gene [46]. The heat map presents the fold change in 2^(−∆∆CT)^ values. The lack of data means there was no mRNA expression detected (Ct cycle > 35). The fold change expression ranged between 0 and 0.20. The 0 value or green color means the level of expression is low, while the 0.20 value or red color means the level of expression is high.

**Figure 2:**
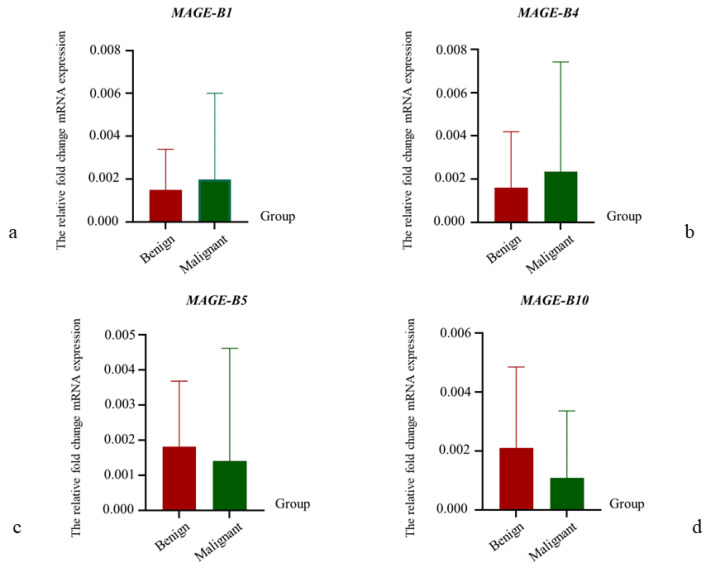
The comparisons of mean level of relative fold change in the mRNA expression results of *MAGE-B1, -B4, -B5, and -B10* genes between benign and malignant CMTs. (**a**): A comparison of the mean level of relative fold change in the mRNA expression results of *MAGE-B1* between benign CMTs (*n* = 4) and malignant CMTs (*n* = 24). (**b**): A comparison of the mean level of relative fold change in the mRNA expression results of *MAGE-B4* between benign CMTs (*n* = 4) and malignant CMTs (*n* = 24). (**c**): A comparison of the mean level of relative fold change in the mRNA expression results of *MAGE-B5* between benign CMTs (*n* = 4) and malignant CMTs (*n* = 19). (**d**): A comparison of the mean level of relative fold change in the mRNA expression results of *MAGE-B10* between benign CMTs (*n* = 3) and malignant CMTs (*n* = 20).

**Figure 3:**
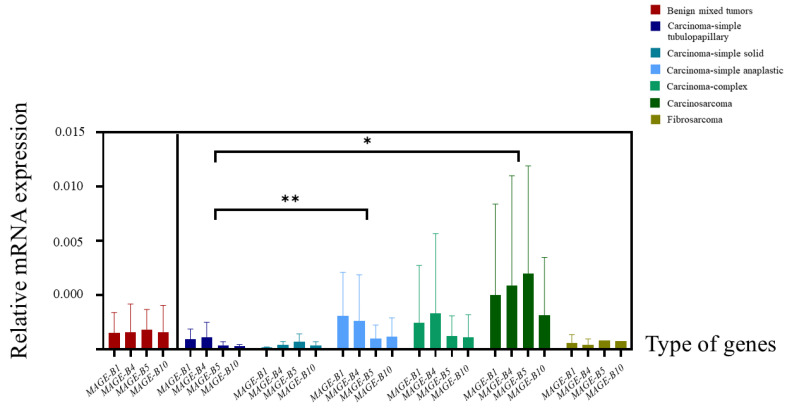
The comparison of the mean relative fold change in the mRNA expression results of *MAGE-B* genes between different histopathological types of canine mammary gland tumor tissue samples using the nested *t*-test analysis [46]. * Different significant values between groups were at *p* ≤ 0.01 and ** different significant values between groups were at *p* < 0.05.

The results of the relative *MAGE-B* mRNA expression were calculated using the specified method and were illustrated separately according to histopathological subtype using a heat map (Figure 1). The mean levels of the relative fold change in the mRNA expression of *MAGE-B* genes were compared between benign and malignant CMTs (Figure 2). In comparisons between groups, the mean levels of the relative fold change in the mRNA expression of *MAGE-B1* and *-B4* were higher in malignant CMTs than in benign CMTs (Figure 2a,b). On the other hand, the mean levels of relative fold change in the mRNA expression of *MAGE-B5* and *-B10* were lower in malignant CMTs than in benign CMTs (Figure 2c,d). Among benign CMTs, the mean level of relative fold change in the mRNA expression of *MAGE-B10* was the highest, followed by *MAGE-B5*, *-B4*, and *-B1*, respectively (Figure 3). Among malignant CMTs, the highest mean level of relative fold change in the mRNA expression of *MAGE-B* genes was observed in carcinosarcoma, followed by carcinoma-complex and carcinoma-simple anaplastic, respectively (Figure 3). Interestingly, the nested t-test analysis showed that the mean level of relative fold change in the mRNA expression of the *MAGE-B* gene group in the carcinosarcoma CMT samples was significantly higher than that in the carcinoma-simple tubulopapillary type of CMT samples (*p* ≤ 0.05). In addition, the relative fold change in the mRNA expression of the *MAGE-B* gene group in the carcinoma-simple anaplastic CMT samples was significantly higher than that in the carcinoma-simple tubulopapillary CMT samples (*p* ≤ 0.01).

### 3.4. The Influence of 5-aza-CdR and TSA on mRNA Expression Relations

**Figure 4:**
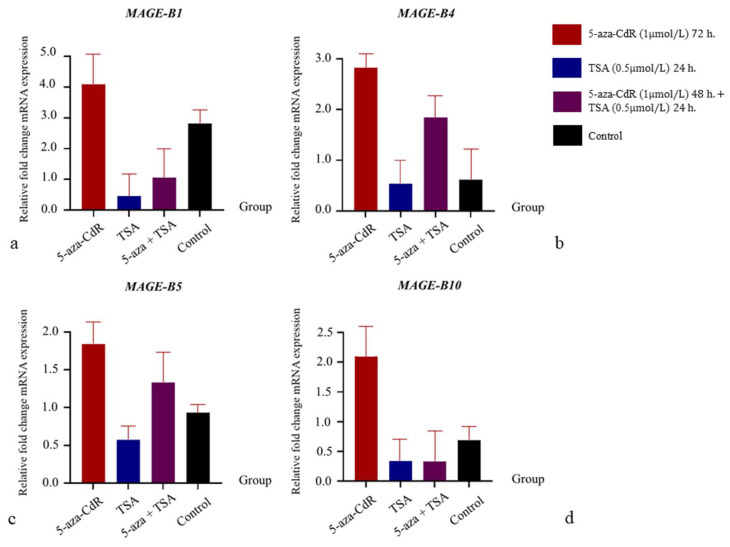
The effects of 5-aza-CdR and/or TSA on the relative fold change in *MAGE-B1*, *-B4*, *-B5*, and *-B10* mRNA expression in the benign type of CMT primary cells. (**a**): The relative fold change in *MAGE-B1* mRNA expression in the benign type of CMT primary cells in different treatments. (**b**): The relative fold change in *MAGE-B4* mRNA expression in the benign type of CMT primary cells in different treatments. (**c**): The relative fold change in *MAGE-B5* mRNA expression in the benign type of CMT primary cells in different treatments. (**d**): The relative fold change in *MAGE-B10* mRNA expression in the benign type of CMT primary cells in different treatments.

**Figure 5:**
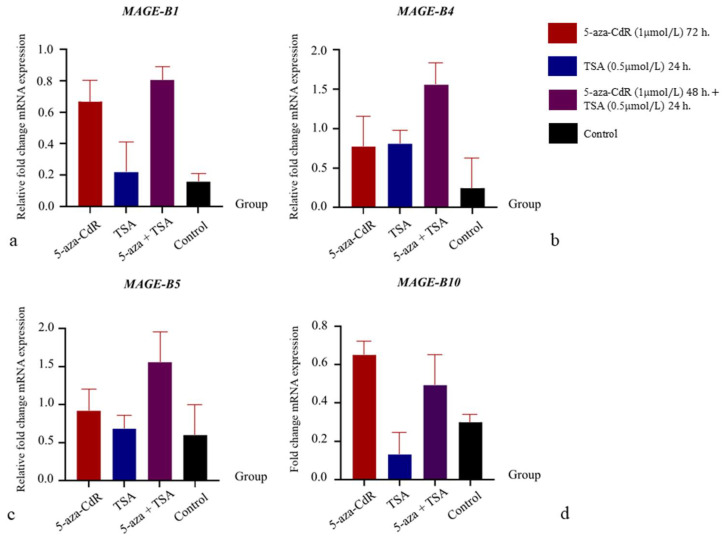
The effects of 5-aza-CdR and/or TSA on the relative fold change in *MAGE-B1*, *-B4*, *-B5*, and *-B10* mRNA expression in the malignant type of CMT primary cells. (**a**): The relative fold change in *MAGE-B1* mRNA expression in the benign type of CMT primary cells in different treatments. (**b**): The relative fold change in *MAGE-B4* mRNA expression in the benign type of CMT primary cells in different treatments. (**c**): The relative fold change in *MAGE-B5* mRNA expression in the benign type of CMT primary cells in different treatments. (**d**): The relative fold change in *MAGE-B10* mRNA expression in the benign type of CMT primary cells in different treatments.

The influence of the DNA methylase inhibitor (5-aza-CdR) and histone deacetylase inhibitor (TSA) on the mRNA expression in benign mixed tumor cells is shown in Figure 4, and that in carcinoma-complex cells is shown in Figure 5. In benign mixed tumor cells, the level of fold change in *MAGE-B1*, *-B4*, *-B5*, and *-B10* mRNA expression in cells treated with 5-aza-CdR was higher than in the control group, while the level of fold change in *MAGE-B1*, *-B4*, *-B5*, and *-B10* mRNA expression in cells treated with TSA was lower than in the control group. Additionally, the level of *MAGE-B1*, *-B4*, *-B5*, and *-B10* mRNA expression in cells treated with 5-aza-CdR followed by TSA was lower than in cells treated with 5-aza-CdR alone. In carcinoma-complex cells, the level of fold change in *MAGE-B1*, *-B4*, *-B5*, and *-B10* mRNA expression in cells treated with 5-aza-CdR or 5-aza-CdR followed by TSA was higher than in the control group. In contrast, the level of fold change in *MAGE-B1*, *-B4*, and *-B5* mRNA expression in cells treated with TSA alone was higher than in the control group, while the level of fold change in *MAGE-B10* mRNA expression in cells treated with TSA alone was lower than in the control group. In contrast to benign mixed tumor cells, the level of fold change in *MAGE-B1*, *-B4*, and *-B5* mRNA expression in malignant cells treated with 5-aza-CdR followed by TSA was higher than in those treated with 5-aza-CdR alone. Moreover, the level of fold change in *MAGE-B10* mRNA expression in malignant cells treated with 5-aza-CdR followed by TSA was lower than in those treated with 5-aza-CdR alone.

## 4. Discussion

The *MAGE-B* family of genes has attracted attention as a potential candidate for targeting cancer immunotherapy, especially in the form of tumor vaccines against MAGE-B antigen-expressed tumor cells after surgery [51]. These proteins are specifically expressed by tumor cells and are displayed on the cell surface as MHC–peptide antigen complexes, alerting the immune system for detection and eradication [28,52]. However, knowledge of these genes has been limited in the field of veterinary oncology. Therefore, the present study developed a qPCR method to explore the mRNA expression of the *MAGE-B* gene family in canine mammary tumor (CMT) tissues and cells, and investigated the role of DNA methylation and histone deacetylation in their regulation.

High-quality total RNA is critical for the success of molecular studies [53]. The present study demonstrated the high quality of total RNA preparation from tissues and cells. Amplification efficacy is also a key parameter in quantifying the effectiveness of the amplification in qPCR assays. According to the results, the *GAPDH*, *MAGE-B1*, *MAGE-B4*, and *MAGE-B10* primers displayed a high efficacy, with amplification efficiencies between 90% and 110%, making them excellent primer pairs [54]. However, designing primers for *MAGE-B5* proved challenging due to the high homology of its sequence with the other *MAGE-B* genes. Despite this, the amplification efficacy of the *MAGE-B5* primer was deemed acceptable, and its specificity was high because the *MAGE-B5* primers were located on two exons. These primers targeted the regions of the gene that encode parts of the final mature mRNA after introns had been removed through RNA splicing [55]. Moreover, these primer pairs were confirmed to have no homology with other canine gene sequences.

This study found that the proportions of *MAGE-B1*, *MAGE-B4*, *MAGE-B5*, and *MAGE-B10* mRNA expressed in CMT tissues were high. A recent study reported a high proportion of *MAGE-B2, MAGE-B3*, *MAGE-B4*, *MAGE-B5*, and *MAGE-B6* gene expression in human colon cancer tissue, with proportions of 70%, 90%, 60%, 50%, and 90%, respectively [56]. In contrast, some studies reported a low expression of the *MAGE-B1* and *-B4* genes, with only 10% (1/10) of breast cancer cells expressing *MAGE-B1*, while no *MAGE-B5* was observed to be expressed in breast cancer cells [57]. Another study found *MAGE-B1* expression in 11% (6/57) of human gastric carcinoma tissues and 17% (9/53) of human esophageal carcinoma tissues [31]. Additionally, *MAGE-B10* expression was observed in colorectal cancer, where it may be associated with carcinogenesis and cell proliferation [58]. Previous studies have suggested that *MAGE-B* knockdown suppresses the growth of mast cell cells in syngeneic mouse tumor models, indicating that *MAGE-B* genes may have oncogenic activity [59]. Furthermore, MAGE-B protein expression in melanoma cell lines may suppress apoptosis by suppressing p53, actively contributing to the development of malignancies and promoting tumor survival [60]. According to these results, the levels of *MAGE-B1*, *-B4*, *-B5*, and *-B10* in both carcinosarcoma and carcinoma-simple anaplastic tumors were higher than those in carcinoma-simple tubulopapillary tumors. These findings may be related to a previous study indicating that dogs with carcinosarcoma and anaplastic sarcoma had a survival time of zero at one year after surgery, while dogs with simple tubulopapillary had a 75% survival rate at one year [61]. However, further studies on *MAGE-B* in tumor progression and prognosis are needed. Moreover, *MAGE-B5* and *-B10* exhibit high expression levels in benign CMT cells, characterized by both the proportion and level of expression; however, moderate expression is reported in malignant cells, especially mesenchymal CMTs, according to the results. These intriguing data should be further investigated in the expressed specific cells using immunohistochemistry with *MAGE-B5* and *-B10* antibodies, exploring their relationship with the microenvironment.

The epigenetic regulation of gene expression in cancer involves two main mechanisms—DNA methylation and histone deacetylation [62]. The present study in dogs aligns with previous studies on *MAGE* family gene expression in various human tumor cells, showing that the DNA methyltransferase inhibitor 5-aza-CdR increases the level of *MAGE-B* mRNA expression in mammary tumor cells [42,63]. As expected, the level of *MAGE-B* mRNA expression in cells treated with 5-aza-CdR increased compared to the untreated group, which is consistent with previous studies conducted in human tumor cells [42,43,63,64]. 5-aza-CdR, a reagent that requires activation via phosphorylation by deoxycytidine kinase, inactivates DNA methyltransferase, leading to DNA hypomethylation in cells [65]. These findings suggest that DNA hypomethylation may drive the expression of *MAGE-B1*, *MAGE-B4*, *MAGE-B5*, and *MAGE-B10* mRNA in primary benign and malignant CMT cells. Likewise, several studies have supported the present findings, showing that tumor cells treated with TSA alone had a reduced mRNA expression, or that the effect was minimal in decreasing the expression of the *MAGE* gene family [45,48,66]. Trichostatin A (TSA) is a potent and specific inhibitor of histone deacetylase (HDAC) activity, which acts as cytostatic, differentiating properties in mammalian cell cultures [67,68]. Previous studies have revealed that this reagent inhibited mammary tumor cell proliferation at the G1-S phase of the cell cycle [67,69]. The tumor cell proliferation alteration due to the TSA reagent may suggest the involvement of the downregulation of *MAGE-B* mRNA expression in CMT cells. Similar to studies on *MAGE-A* genes in human tumors, the present study revealed that the addition of TSA after 5-aza-CdR treatment further increased the induction of *MAGE-B1*, *-B4*, and *-B5* mRNA expression in the carcinoma-complex type of CMT cells [41,43,44,48]. This suggests a synergistic effect in activating *MAGE-B* expression, where DNA hypomethylation drives expression from a silent state, and histone deacetylation may act as a subsequent event. Despite these findings, no synergistic effect was observed for *MAGE-B10* expression in our study. This remains a controversial topic, as some studies have also found a lack of *MAGE-B* expression in human cells [48,66]. The concentration of TSA reagents may play a role, as high DNA methyltransferase activity or inadequate levels of transcription factors may be involved in this discrepancy [48]. However, our study was preliminary; a larger cohort with expansion to other types of canine tumors and normal tissues would strengthen the conclusions and provide more reliable data on expression patterns, warranting further investigation.

## 5. Conclusions

The proportion of *MAGE-B1*, *-B4*, *-B5*, and *-B10* expression in CMT tissue samples was high in both benign and malignant CMTs, with their expression levels ranging from 82.14 to 100.00% across all samples. Moreover, this study highlighted that carcinosarcoma and carcinoma-simple anaplastic tumors have significantly higher levels of mRNA expression compared to carcinoma-simple tubulopapillary CMTs. Additionally, the study found that DNA hypomethylation may drive the expression of *MAGE-B1*, *-B4*, *-B5*, and *-B10* mRNA in CMT cells. Furthermore, a larger cohort would strengthen the conclusions and provide more reliable data on expression patterns, warranting further investigation.

## Figures and Tables

**Table 1:** Primers.

No.	Genes	Specific Primers	Size (bp)	NCBI Database	Efficiency	R^2^
1	*GAPDH*	5′-GTCATCATCTCTGCTCCTTCTG-3′	90	NM_001003142	0.90	0.99
		5′-GCTGACAATCTTGAGGGAGTT-3′				
2	*MAGE-B1*	5′-GCCTCTTCTGAGTGTGATCTTC-3′	104	XM_005641221	0.92	0.98
		5′-CTACGTGCTTCCTTCCTTCATAG-3′				
3	*MAGE-B4*	5′-CATGAGCACCCTAAACCTC-3′	108	XM_005641220	0.93	0.97
		5′-TTGCCATTCAAGAAGATCACAC-3′				
4	*MAGE-B5*	5′-TGAGATCCTTAAGCAAGCCT-3′	119	XM_014111462	0.88	0.99
		5′-CATTGTTGGGTAGCTTCACT-3′				
5	*MAGE-B10*	5′-GCTGGGTCATATAGCGTTTC-3′	108	NM_001003116	0.98	0.97
		5′-CACTTGGTTGTCAGCACTTTC-3′				

**Table 2:** The proportion of *MAGE- B1, -B4, -B5, and B-10* mRNA expression in canine mammary gland tumors (N = 28).

Type of Tumors (Number of Samples)			Age (Mean ± S.D.)	Proportion of *MAGE-B* mRNA Expression (%)			
				*MAGE-B1*	*MAGE-B4*	*MAGE-B5*	*MAGE-B10*
Benign (4)			10.67 ± 1.92	4/4 (100)	4/4 (100)	4/4 (100)	4/4 (100)
Malignant (24)			10.21 ± 2.15	24/24 (100)	24/24 (100)	79.2 (19/24)	17/24 (70.8)
	Epithelial neoplasms (19)		10.04 ± 2.08	19/19 (100)	19/19 (100)	16/19 (84.2)	16/19 (84.2)
		Carcinoma-simple (13)	9.08 ± 1.88	13/13 (100)	13/13 (100)	12/13 (58.3)	10/13 (76.9)
		Carcinoma-complex (6)	9.73 ± 1.07	6/6 (100)	6/6 (100)	4/6 (71.4)	6/6 (100)
	Carcinosarcoma (3)		11.67 ± 2.51	3/3 (100)	3/3 (100)	2/3 (75.0)	3/3 (100)
	Mesenchymal neoplasms (2)		12.50 ± 3.53	2/2 (100)	2/2 (100)	1/2 (50)	1/2 (50)
Total CMT (28)			10.39 ± 2.13	28/28 (100)	28/28 (100)	23/28 (82.1)	24/28 (85.7)

## Data Availability

The data presented in this study are available on request from the corresponding author due to the policy of the veterinary teaching hospital.
